# HISTORY OF CAREGIVING SURVEILLANCE AT THE CDC, 2005 TO PRESENT

**DOI:** 10.1093/geroni/igz038.674

**Published:** 2019-11-08

**Authors:** Erin D Bouldin

**Affiliations:** Appalachian State University, Boone, North Carolina, United States

## Abstract

Research on caregiving has been ongoing for decades, but little systematic data collection at the population level occurred until relatively recently. Surveillance data is critical because it provides an evidence base upon which to make informed decisions about allocating resources, targeting programs, and developing policy. In 2005, CDC and the Association for Prevention Teaching and Research funded Dr. Elena Andresen’s proposal to develop a set of questions about caregiving to be used on the BRFSS. This Caregiver Module was piloted in North Carolina and has been used – with some modifications – ever since as an optional module. The Caregiver Module has provided state-level data on the prevalence and types of caregiving provided by community-dwelling adults age 18 and older to people with a variety of conditions and needs. In 2009, the caregiver screening question was included on the BRFSS core, yielding a national estimate of caregiving prevalence of 24.7%.

